# Supplementary material for: The humanitarian-development nexus and sexual and reproductive health interventions in fragile settings: A scoping review
Source: PLOS Glob Public Health. 2026 Feb 4;6(2):e0005767. doi: 10.1371/journal.pgph.0005767 (PMC12871988; doi:10.1371/journal.pgph.0005767)
Supplement: S2 Text — (DOCX) [file pgph.0005767.s004.docx]

**S2 Text. Grey Literature Sources**

| - ReliefWeb |
| --- |
| - HumanitarianResponse.info |
| - Inter-Agency Working Group on Reproductive Health in Humanitarian Crises (IAWG) |
| - USAID Development Experience Clearinghouse (DEC) |
| - USAID Nutrition Resource Hub |
| - Healthy Newborn Network |
| - Child Health Task Force |
| - FP2030 |
| - CORE Group |
| - Implementing Best Practices (IBP) Network |
| - Maternal and Child Survivor Program (MCSP) |
| - Maternal and Child Health Integrated Program (MCHIP) |
| - Knowledge Success |
| - Inter-Agency Standing Committee (IASC) |
| - The World Bank |
| - United Nations websites: WHO, UNFPA, OCHA, UNICEF, UNHCR |
